# Bathing Suit Variant of Autosomal Recessive Congenital Ichthyosis (ARCI) in Two Indian Patients

**DOI:** 10.1155/2018/3140473

**Published:** 2018-12-30

**Authors:** Dharshini Sathishkumar, Dincy Peter, Susanne Pulimood, Henning Wiegmann, Frederic Valentin, Meera Thomas, Hans Christian Hennies, Vinzenz Oji

**Affiliations:** ^1^Department of Dermatology Venereology and Leprosy, Christian Medical College, Vellore, India; ^2^Department of Dermatology, University Hospital Muenster, Muenster, Germany; ^3^Center for Dermatogenetics, Division of Human Genetics, Medical University of Innsbruck, Innsbruck, Austria; ^4^Cologne Center for Genomics, Division of Dermatogenetics, University of Cologne, Germany; ^5^Cologne Excellence Cluster on Cellular Stress Responses in Aging-Associated Diseases (CECAD), University of Cologne, Cologne, Germany; ^6^Department of Biological and Geographical Sciences, University of Huddersfield, Huddersfield, UK

## Abstract

Bathing suit ichthyosis (BSI) is a rare variant of autosomal recessive congenital ichthyosis (ARCI) due to transglutaminase-1 gene (*TGM1*) mutations leading to a temperature sensitive phenotype. It is characterized by dark-grey or brownish scaling restricted to the “bathing suit” areas. We report two Indian girls with bathing suit ichthyosis and mutations in* TGM1 *(patient 1: homozygous for c.1147G>A; patient 2: compound heterozygous for c.832G>A, c.919C>G).

## 1. Introduction

Bathing suit ichthyosis (BSI) is a rare variant of lamellar ichthyosis due to transglutaminase-1 gene (*TGM1*) mutations leading to a temperature sensitive phenotype [1]. First case was described in 1972 by Scott [2], followed by a case series by Jacyk, both from South Africa [3]. A current study by Marukian et al. expanded the group of reported BSI cases by additional 9 new mutations in patients from different ethnic origins [4]. Altogether, the majority of BSI reports are from Africa, Europe, Turkey, Middle East, and China but no reports from India so far. We report two cases of unrelated Indian girls with bathing suit ichthyosis and confirmatory mutations in* TGM1*.

## 2. Case Report

**Figure 1:**
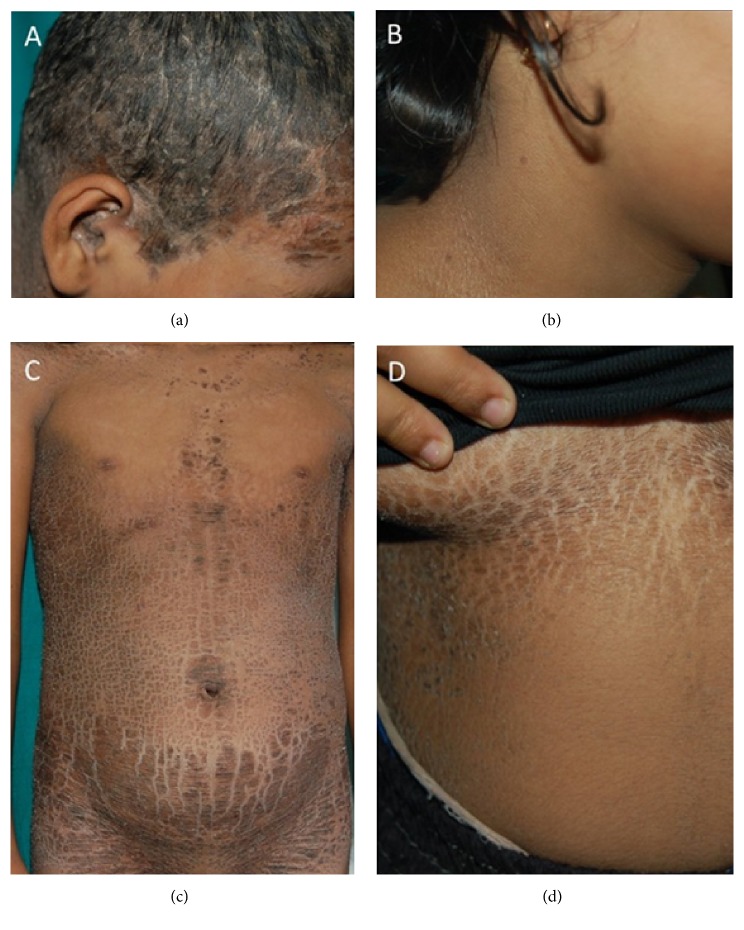
**Clinical presentation of patient 1 and 2**. (a, c). Patient 1 shows a lamellar type of ichthyosis involving the forehead, external auditory meatus, and neck, but sparing the centrofacial area by additional involvement of the bathing suit area. (b, d) Patient 2 shows a mild ichthyosis confined to bathing suit area with involvement of the axilla, but sparing of ears and face. The patient has been seen in winter.

**Figure 2:**
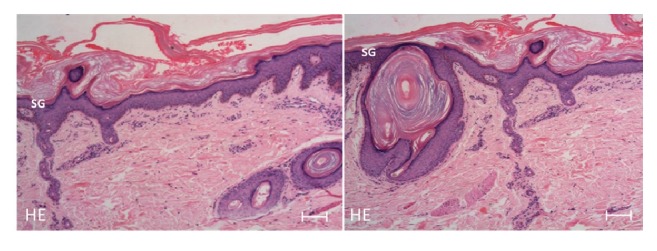
Histopathology of the ichthyotic area in patient 2 shows hyperkeratosis with follicular plugging and hyperplasia of the epidermis with well-preserved stratum granulosum. (*HE*: haematoxylin & eosin;* SG*: stratum granulosum; scale bare = 100 *μ*m).

Two unrelated girls aged nine (patient 1) and 12 (patient 2) years born from nonconsanguineous parents presented at birth with collodion membranes and generalized scaling. The collodion resolved and left behind erythroderma in the neonatal period. At the age of three months patient 1 developed ichthyosis confined to the trunk. In patient 2 this phenotypic shift developed at the age of three years. Both had history of summer exacerbation and improvement in winter. Interestingly the first patient had history of erythroderma during episodes of fever. No members of their family or pedigree had similar symptoms. They had no past history of serious diseases other than ichthyosis. On examination, patient 1 had large brownish, lamellar scales involving the bathing suit area of the trunk including the neck, forehead, axillae, external auditory meatus, and thick scales on the scalp (Figures 1(a) and 1(c)). The centrofacial region was spared. In addition, she had focal plantar keratoderma. There was marked reduction of scaling during the cold season. Patient 2 was seen in winter, and she had mild ichthyosis confined to the bathing suit area (Figures 1(b) and 1(d)). Skin biopsy from her ichthyotic area showed hyperkeratosis with follicular plugging, mild hyperplasia of the epidermis with well-preserved granular layer, no parakeratosis, and patchy perivascular infiltrates of lymphohistiocytes (Figure 2). The diagnosis of bathing suit ichthyosis (BSI) was made in both patients based on the criteria proposed by Jacyk et al. [3]. The patients were treated with bland ointments and mild keratolytics such as urea cream.

## 3. Results

**Figure 3:**
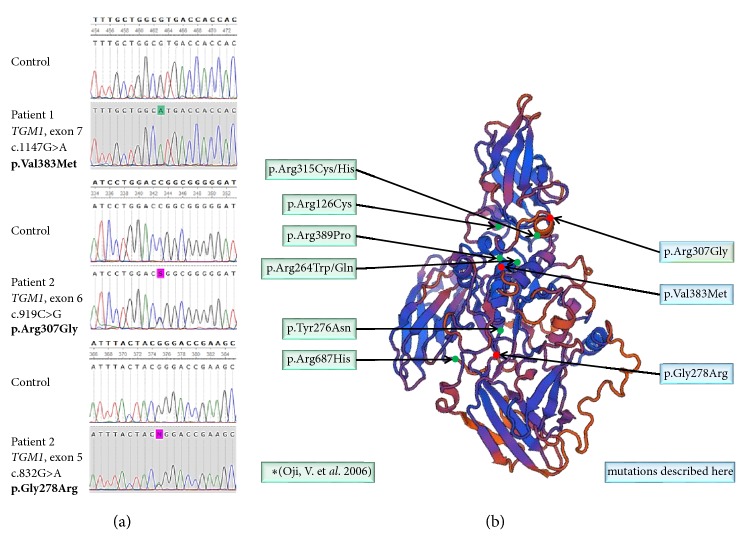
**Localization of mutations within transglutaminase 1 causing bathing suit ichthyosis**. (a) Results of sanger sequencing of patient 1 and 2 to confirm mutations described here. (b) The localization of the 2006 by Oji et al. published mutations p.Arg315Cys/His, p.Arg126Cys, p.Arg389Pro, p.Arg264Trp/Gln, p.Tyr276Asn, p.Arg687His, p.Arg307Gly (Oji, V. et al. 2006), and the two mutations p.Val383Met and p.Gly278Arg found in two Indian children is depicted within a three-dimensional model of transglutaminase 1. The well-known mutation p.Arg307Gly was also found in the two Indian children. Three-dimensional modeling of transglutaminase 1 isoform 1 was performed by using the online* in-silico* protein structure prediction tool SWISS MODEL (Swiss institute of Bioinformatics, SIB). For structural alignment the structure of transglutaminase 3 was used as template. (b) Results of sanger sequencing of patient 1 and 2 to confirm mutations described here.

DNA of each patient has been extracted from full blood and analyzed for mutations in* TGM1* by direct Sanger sequencing of all coding exons and flanking intronic sequences. Patient 1 was homozygous for the mutation c.1147G>A in exon 7, which leads to an exchange of valine by methionine, p.Val383Met. Patient 2 was compound heterozygous for the two* TGM1 *mutations c.832G>A in exon 5 and c.919C>G in exon 6. These DNA changes lead to the missense mutations p.Gly278Arg and p.Arg307Gly, respectively. The aforementioned changes of the amino acids are visualized within a 3-dimensional protein model of TGM1 (Figure 3).

## 4. Discussion/Conclusion

Bathing suit ichthyosis (BSI) is a rare phenotypic variant of autosomal recessive congenital ichthyosis (ARCI). Patients are born as collodion babies; after shedding of the collodion membrane, large dark grey or black scales develop but are confined to the bathing suit areas. Approximately 50 cases have been reported so far [1, 4–7]. Even though initially thought to be a South African genodermatosis [8] an increasing number of cases have been reported from other parts of the world [1, 2, 4–6, 8], and this report represents one of the first genetic studies on BSI in Indian patients. Both our patients had characteristic features of BSI. An important diagnostic clinical sign is the involvement of warmer skin areas such as the neck or the excessive desquamation of the external auditory meatus, which required repeated syringing [1, 6].

BSI is caused by transglutaminase-1 deficiency with heat-dependent transglutaminase-1 dysfunction [1, 7]. The enzyme plays a major role in the formation of the cornified cell envelope (CE) by cross-linking of CE precursor proteins and ceramides. Transglutaminase-1 deficiency is supposed to lead to a defective cornified cell envelope formation and a disturbance of the lipid barrier.

Specific mutations of* TGM1* lead to reduced transglutaminase-1 activity at higher temperature [7], which explains the summer exacerbation and fever induced erythroderma seen in patient 2 [6]. Moreover, it is speculated that hypohidrosis following excessive keratinization can result in heat accumulation, which possibly causes additional hyperkeratosis in the summer, and that this vicious circle may contribute to the seasonal variation in BSI [4]. The homozygous missense mutation p.Val383Met, which was identified in patient 1, has been described in other patients with BSI [5] and in one case of lamellar ichthyosis, which would be regarded as a case of BSI retrospectively [6]. The* TGM1* mutation p.Arg307Gly of patient 2 is known from the original BSI series of Oji et al., 2006 [1]. The second mutation p.Gly278Arg found in the compound heterozygous patient 2 was described as a mutation associated with typical lamellar ichthyosis [9]. This observation corroborates the assumption that one temperature responsive mutation in* TGM1* in a compound heterozygous case is sufficient to give rise to the phenotype of BSI [1].

In summary, we confirm that bathing suit ichthyosis can be found in India and is caused by specific mutations in* TGM1*. Clinicians should be aware of the clinical key features of BSI, especially the heat sensitivity of this special ichthyosis variant representing the diagnostic clue. Of note, the refinement of genotype-phenotype correlation within the* TGM1* gene mutation spectrum may improve the counselling situation and management of families with collodion babies.
